# Mobile apps for cancer patient education: a technology foresight analysis

**DOI:** 10.1007/s00520-026-11112-z

**Published:** 2026-08-19

**Authors:** Bruna de Castro Ornellas, Luciana Regina Ferreira da Mata, Bruno Marques Silva, Elaine Barros Ferreira, Simone Roque Mazoni, Christiane Inocencio Vasques

**Affiliations:** 1 Master and Doctorate in Nursing Program, School of Health Sciences, University of Brasilia, Brasília, DF Brazil; 2 School of Nursing, Federal University of Minas Gerais, Belo Horizonte, MG Brazil; 3 Department of Nursing, School of Health Sciences, University of Brasilia, Brasília, DF Brazil; 4 University of Brasilia, School of Health Sciences, Campus Universitário Darcy Ribeiro, Asa Norte, Brasília, DF 70910-900 Brazil

**Keywords:** Digital health, Mobile applications, Patient education, Oncology, Nursing, MHealth

## Abstract

**Purpose:**

To identify and classify free mobile applications for adult cancer patient education available in the App Store and Google Play.

**Methods:**

A technology foresight study was conducted in four phases (preparation, pre-prospecting, prospecting, and post-prospecting). Search was carried out in both stores, using the terms “cancer” and “oncology”. Free apps in English, Spanish, or Portuguese aimed at educating adults with cancer were included. Quality was assessed based on a scale adapted from the Applications Scoring System.

**Results:**

A total of 848 apps were identified. After screening, 32 met the inclusion criteria. Most were categorized under “Medicine” (*n* = 21), were available in the App Store (*n* = 25), and were released between 2017 and 2024, peaking in 2024 (*n* = 14). Content most frequently addressed breast cancer. Only nine apps achieved the maximum app comprehensiveness score, and seven cited scientific literature. Most required internet connectivity (*n* = 28) and were easy to navigate (*n* = 29), whereas professional validation and offline resources were limited. Overall quality scores ranged from 5 to 10, with a median score of 8 (interquartile range [IQR]: 6–9).

**Conclusion:**

Although digital solutions for oncology education have expanded rapidly, a substantial proportion of available applications remain devoid of a rigorous scientific foundation and continue to exhibit significant barriers to digital accessibility. The development and dissemination of inclusive, evidence-based, and clinically validated digital tools are therefore essential to ensure their effectiveness, reliability, and equitable adoption in oncology education.

## Introduction

Digital transformation has significantly impacted healthcare, driving the emergence of digital health. Digital health technologies (DHT) encompass computing platforms, software, connected devices, sensors, and resources such as mobile applications (apps), wearable devices, video conferencing tools, chatbots, artificial intelligence (AI), and virtual reality (VR) [1, 2].

In oncology, these technologies enable the collection and processing of large data volumes, supporting personalized treatments and patient-centered care. Apps and other digital solutions assist with shared decision-making, monitoring of symptoms and adverse events, communication, emotional support, and health education [2].

The use of smartphones and tablets is growing among the general population. In 2024, an estimated 60.42% of the global population were smartphone users, which represents 4.88 billion people [3]. By 2020, approximately 350,000 digital health apps were available in commercial stores, supporting symptom tracking, medication adherence, access to test results, self-management, and patient empowerment [2, 4, 5].

Interventions mediated by mobile technologies have demonstrated effectiveness in supporting self-care and enhancing patient engagement [5]. Adoption in oncology has accompanied the transition from exclusively in-person models to digital approaches capable of providing continuous, real-time assessment and intervention [6, 7]. DHT have the potential to strengthen communication between users and health-care professionals, improve monitoring of symptoms and adverse effects, and promote active engagement throughout cancer treatment [6, 7].

Over the past four decades, provision of information has been associated with reduced patient anxiety, although reports of dissatisfaction with the guidance received persist [2]. When applied to health education, DHT may increase user satisfaction and improve the patient experience, with potential to reduce morbidity and mortality from cancer-related complications and to lower costs from avoidable readmissions and hospitalizations. The positive effect of DHT-mediated education derives from facilitating recognition and monitoring of warning signs, enabling earlier intervention [2].

Initiatives such as “Chatbot Vik,” developed to support breast cancer patients, have shown effectiveness comparable to that of physicians in providing information [8]. A review of online educational programs for men with prostate cancer reported increased knowledge and improved satisfaction during treatment [2, 9]. A randomized clinical trial evaluated the effectiveness of symptom management using an advanced remote system for cancer patients. Participants reported a greater sense of safety and encouragement with the guidance provided by digital technology and incorporated the system into daily routines, although anxiety increased when devices were withdrawn at study end [10].

Despite this potential, adoption of DHT faces barriers related to inequalities in digital health literacy, access to technology, connectivity, and infrastructure, which can exacerbate inequities in cancer care [11, 12]. Considering cultural preferences, social determinants of health, and levels of health and digital technology literacy is therefore essential in development and implementation [13–15].

Previous literature reviews have evaluated mobile health apps across different clinical contexts. Overall, the evidence indicates rapid growth in available apps alongside concerns regarding information quality, transparency of sources, and involvement of health professionals in development and evaluation [16–18]. Taken together, these findings demonstrate the need for critical and comprehensive analyses. Accordingly, this study aimed to identify and classify free mobile apps for adult cancer patient education available in the App Store and Google Play.

## Materials and methods

### Study design

A technology foresight study was conducted to map mobile apps aimed at educating cancer patients, available on Android (Google Play®) and iOS (App Store®). Technology foresight enables systematic analyses to identify, map, and anticipate emerging technological trends that may affect specific sectors or the development of new products and services [19, 20]. The guiding question of this study was: Which mobile apps available for Android and iOS target cancer patient education, and what are their main technological and educational features?

### Technology foresight study phases

Technology foresight studies are typically organized in four phases: (1) preparation (definition of objectives, scope, methodological approach, and techniques); (2) pre-prospecting (refinement of methods and identification of data sources); (3) prospecting (collection, processing, and analysis of information retrieved in the searches); and (4) post-prospecting (dissemination of results) [20].

## Preparatory phase

During the preparatory phase, the study objective, research question, study scope, search platforms (Google Play Store® and Apple App Store®), search procedures, application eligibility criteria, and quality assessment instrument were defined.

A study protocol was subsequently developed, detailing the eligibility criteria, data extraction variables, and application quality assessment strategy.

The research protocol was reviewed by the members of the research team, composed of four PhD nurses, including one with specific expertise in mobile app development and validation, to ensure content relevance and methodological rigor. After protocol review, searches were performed in the App Store® and Google Play® using the keywords “cancer” and “oncology.”

### Eligibility criteria

The study included free mobile apps designed to educate adult cancer patients, available in English, Spanish, or Portuguese. Apps were excluded if they lacked its description in store; were developed for conferences; targeted health professionals; were designed solely for patient-to-patient interaction (social media); targeted children with cancer and their families; or presented technical failures during operation.

For the purposes of this research, a mobile app was defined as any software system made available on a smartphone and used to disseminate skills and knowledge [21]. Health education followed the WHO definition as any combination of learning experiences designed to help individuals and communities improve their health by increasing knowledge, influencing motivation, and improving health literacy [1].

## Pre-prospective phase

During the pre-prospective phase, the search strategy was refined and standardized, and the study data sources were confirmed. The Google Play Store® and Apple App Store® were selected because they are the world's two largest mobile application distribution platforms, encompassing the vast majority of applications available for the Android and iOS operating systems.

A pilot search was conducted using the equivalent English (cancer and oncology), Portuguese (câncer and oncologia), and Spanish (cáncer and oncología) terms to evaluate whether search language affected applications retrieval. As the Portuguese and Spanish terms did not identify any additional eligible applications, the final search strategy used the English keywords only, ensuring a standardized and reproducible search across both app stores.

Searches were performed directly within the Google Play Store® and Apple App Store® using the same search strategy on both platforms.

## Prospective phase

The prospective phase comprised the collection, processing, and analysis of information retrieved in the searches.

### Data collection procedure

Searches in the App Store® and Google Play® were carried out on 5 January 2025 in three phases, using two devices: iPhone 13 (Apple®) and Samsung Galaxy A71 (Samsung®). In Phase 1, one researcher (BCO) screened app titles and descriptions for each keyword and removed duplicates. In Phase 2, two researchers (BCO, BMS) independently reviewed titles and descriptions against the eligibility criteria. In Phase 3, apps selected in Phase 2 were downloaded and evaluated again according to the eligibility criteria, independently, by the same two researchers (BCO, BMS). Disagreements in Phases 2–3 were resolved by a third researcher (CIV). All apps that met the eligibility criteria proceeded to quality assessment.

### Data extraction

Data from included apps were collected using a structured form capturing app name, developer, category, target audience, subject, language, year of release, and user assessment. Variable definitions followed a previously published standardized extraction framework for mobile app analyses [16].

### Quality assessment

App quality was evaluated using an adapted Applications Scoring System [22]. The tool assesses usefulness, functionality, basis on scientific evidence, and ease of navigation, and was initially developed for assess obstetrics and gynecology apps by the Department of Obstetrics, Gynecology and Reproductive Sciences, Icahn School of Medicine at Mount Sinai, New York [16, 23]. The evaluation comprises 13 objective criteria scored binary (0/1) plus a one app comprehensiveness criterion scored 0–3, yielding a 0–16 total (higher scores indicate greater suitability and usefulness in providing health care). For this study, the retained criteria were app comprehensiveness, literature cited, in-app purchases, connectivity, advertisements, search field, cross-device compatibility, images/figures, videos, special features, and ease of navigation. Price was not assessed (exclusion criteria), and the subjective presentation item was omitted. The presentation domain was excluded because it primarily evaluates aesthetic aspects of the user interface rather than the educational or functional characteristics addressed in the objectives of this study.

For comprehensiveness score, apps enabling message exchange with the health service and/or a chatbot, a diary for recording signs and symptoms, and options to share information with others or on social media were considered. Each available feature received one point, resulting in a comprehensiveness score ranging from 0 to 3. For special features, apps offering technical support and/or notification/alarm settings were considered. Thus, the adapted scale evaluated 11 binary items (0 = no; 1 = yes) plus the comprehensiveness score [16]. The evaluation criteria are shown on Table 1.

**Table 1:** Criteria for app evaluation according to score and description

Component	Score	Description
1. App comprehensiveness	3	1 point for each coverage measure
2. Literature used	1	0 (no references), 1 (references used)
3. In-app purchase	1	0 (present), 1 (absent)
4. Connectivity	1	0 (internet required), 1 (internet not required)
5. Advertisements	1	0 (present), 1 (absent)
6. Text search field	1	0 (no search field), 1 (search field present)
7. Compatibility	1	0 (iPhone or Android), 1 (iPhone and Android)
8. Images/figures	1	0 (present), 1 (absent)
9. Videos	1	0 (present), 1 (absent)
10. Special features	1	0 (present), 1 (absent)
11. Ease of Navigation	1	0 (present), 1 (absent)

### Data synthesis and analysis

Data from the included apps were analyzed descriptively and summarized in tables and figures, accompanied by a narrative synthesis. The application quality assessment score was summarized using the median and interquartile range (IQR), given its ordinal and discrete nature and limited number of possible values.

## Post-prospective phase

The post-prospective phase comprised the dissemination of the study findings through the preparation and submission of the findings for scientific publication.

### Ethical aspects

This study analyzed data in the public domain and involved no human participants; therefore, review by a research ethics committee/institutional review board was not required.

## Results

**Fig. 1:**
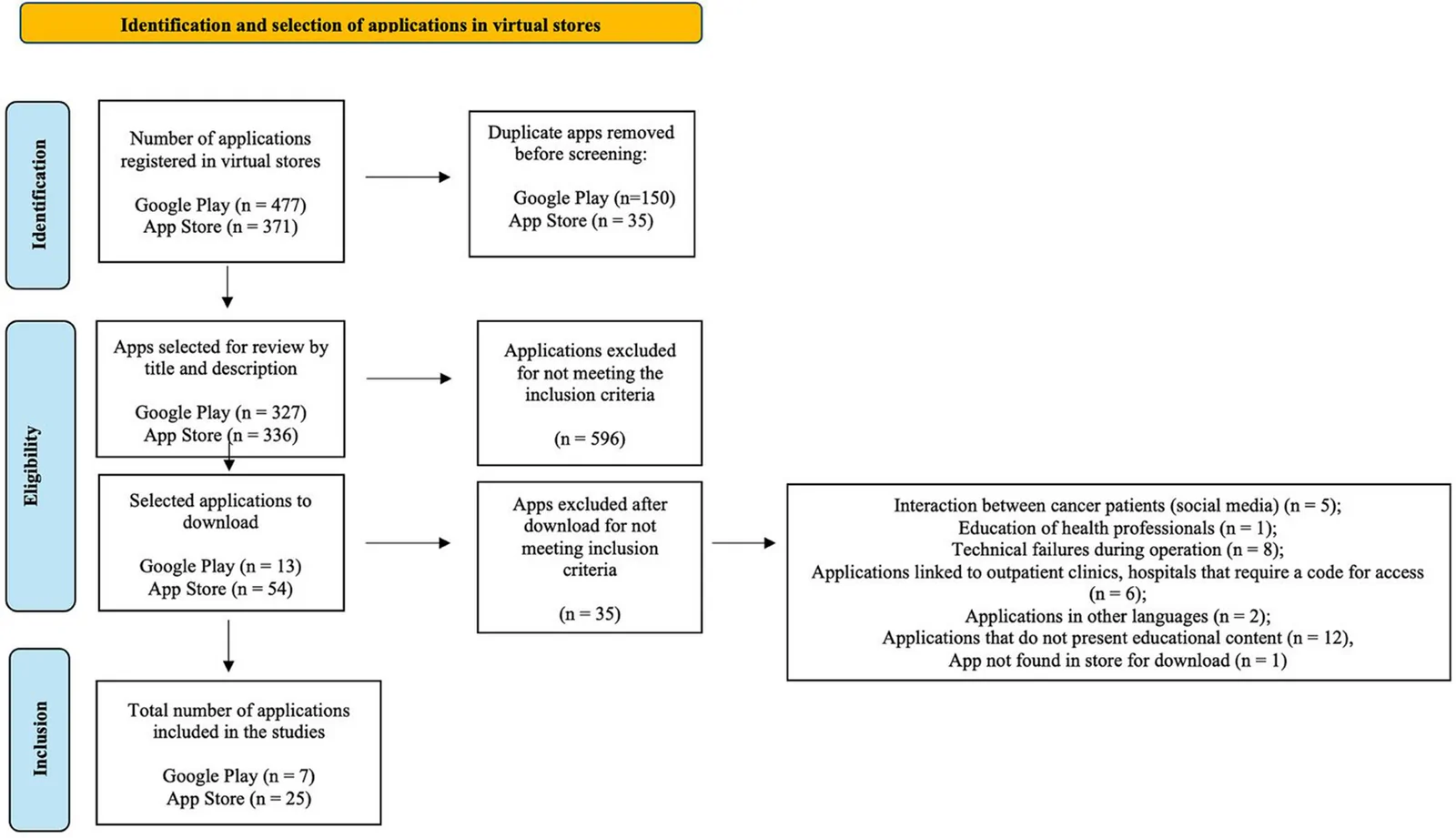
Flowchart of search, identification, and selection of applications for evaluation and reasons for exclusion. Adapted from Prisma, 2020 and Machado et. al., 2022

Searches in the App Store® and Google Play® identified 848 apps. After removing duplicates, 663 unique records remained; 596 were excluded at phase 1 screening. Sixty-seven apps were downloaded for full assessment, of which 35 did not meet the eligibility criteria. The final sample comprised 32 apps (Fig. 1).

A total of 32 mobile apps for cancer patient education were analyzed for trends and characteristics relevant to applicability. Of these, 25 were available in the App Store®, and seven of them were also available on Google Play. Among the seven apps available on Google Play, one app was also available on the App Store. Release year was identified for 31 of the 32 apps and ranged from 2017 to 2024. Of these, 14 (45,16%) were launched in 2024.

Regarding categorization, most apps were listed under “Medicine” (*n* = 21), followed by “Health & Fitness” (*n* = 7); additional categories included “Lifestyle” (*n* = 2), “Social Media” (*n* = 1), and “Business” (*n* = 1). As to target audience, of the 32 apps analyzed, only two were developed exclusively for women, focused on breast and cervical cancer, whereas the remainder, including other breast cancer–related apps, were designed for both sexes. The characteristics of the analyzed apps, organized according to their identification variables, are summarized in Table 2.

**Table 2:** Description of applications according to identification variables

Code	Name	Developer	Category	Target Audience	Subject	Language	Year of publication	User assessment
App 1	Ankr	Ankr Health Company	Health and Fitness	Men and women	Lifestyle and self-care in breast cancer	English	2018	No
App 2	Bcan Bladder Cancer App	Bladder Cancer	Medicine	Men and women	General information about bladder cancer	English	2017	No
App 3	Becca—Breast Cancer Support	Breast Cancer Care	Health and Fitness	Men and women	Lifestyle and self-care in breast cancer	English	2022	Yes
App 4	Bladder Cancer Manager	@Point of care	Medicine	Men and women	General information about bladder cancer	English	2017	No
App 5	Breast Cancer Manager	@Point of care	Medicine	Men and women	General information about breast cancer	English	2024	No
App 6	Breast Cancer Navigator	Mu Sigma Analytics INC	Medicine	Men and women	General information about breast cancer	English	2023	No
App 7	Cancer AI	Taqtik Health, Inc	Medicine	Men and women	General information about different types of cancer treatments	English	2020	No
App 8	Cancer Connection	Allina Health System	Medicine	Men and women	General information about breast cancer	English	2024	No
App 09	Breast Cancer App	Inovamedx	Lifestyle	Men and women	General information about breast cancer	Portuguese	2023	Yes
App 10	Cancergo	Haploscope Inc	Social media	Men and women	General Information about Cancer and Community and Social Support	English	2023	No
App 11	Ceci	Wecancer Technological Solutions	Medicine	Men and women	General information about cancer, cancer treatments, and self-care	Portuguese	2018	Yes
App 12	Endometrial Cancer Manager	@Point of care	Medicine	Men and women	General information about endometrium cancer	English	2024	No
App 13	Head and Neck Cancer Manager	@Point of care	Medicine	Men and women	General information about head and neck cancer	English	2024	No
App 14	Kidney Cancer Manager	@Point of care	Medicine	Men and women	General information about kidney cancer	English	2024	No
App 15	Liver Cancer Manager	@Point of care	Medicine	Men and women	General information about liver cancer	English	2024	No
App 16	NCCN Patient Guides For Cancer	National Comprehensive Cancer Network	Medicine	Men and women	Information on various types of cancer, diagnosis, treatment and management of toxicities	English	2019	No
App 17	Outcomes4me Cancer Care	Outcomes4me Inc	Health and Fitness	Men and women	General information about breast cancer, community and social support	English	2024	No
App 18	Ovarian Cancer Manager	@Point of care	Medicine	Men and women	General information ovarian breast cancer	English	2024	No
App 19	Prostate Cancer Manager	@Point of care	Medicine	Men and women	General information about prostate cancer	English	2024	No
App 20	Rise Against Cancer	Indian Cancer Society	Health and Fitness	Men and women	General information about cancer, community, and social support	English	-	No
App 21	Skin Cancer Manager	@Point of care	Medicine	Men and women	General information about breast cancer	English	2024	No
App 22	Stomach Cancer Manager	@Point of care	Medicine	Men and women	General information about stomach cancer	English	2024	No
App 23	Surviving Breast Cancer Org	Survivingbreastcancer.org, inc	Lifestyle	Men and women	Lifestyle and self-care in breast cancer	English	2022	No
App 24	The Cancer App	Interactive Pharma Solutions Limited	Health and Fitness	Men and women	Information about different types of cancer, treatment and management of toxicities	English	2021	No
App 25	Vann: By Cancer Survivor	Vann	Health and Fitness	Men and women	General information about the treatment of various types of cancer, toxicities, and management of some toxicities	English	2023	No
App 26	Acs Reach	Chronus LLC	Business	Women	General information about cancer	English	2020	No
App 27	Belongai Dave—Cancer Mentor	Belongtail	Medicine	Men and women	General information about cancer, treatment and management of toxicities	English	2024	No
App 28	Breast Advocate	Toliman Health	Medicine	Men and women	General information about breast cancer	English	2018	No
App 29	Cervical Cancer Guide	Apps For Everyone	Medicine	Women	General information about cervix cancer	English	2024	No
App 30	Colon Cancer Guide	Apps For Everyone	Medicine	Men and women	General information about colorectal cancer	Spanish	2024	No
App 31	Xemio—Breast Cancer	Fundación Internet Salud Y Sociedad	Medicine	Men and women	General information about breast cancer and toxicity management	Spanish	2021	No
App 32	Zenonco Cancer Care	Zenonco.IO Cancer Care	Health and Fitness	Men and women	General information about cancer and toxicity management	English	2022	No

Source: Prepared by the authors

Regarding thematic focus, most apps targeted health education for patients with breast cancer (*n* = 10). The remaining apps addressed multiple cancer types, such as head and neck, bladder, kidney, endometrial, ovarian, cervical, stomach, liver, prostate, colorectal, and skin cancer, covering treatment information, toxicity management, self-care, and social support.

Table 3 presents the app scores obtained using the adapted Applications Scoring System. Overall quality scores ranged from 5 to 10, with a median of 8 (IQR, 6–9). A total of 7 apps received the maximum score of 10, whereas 10 apps scored 6 or lower. Regarding comprehensiveness score, 9 apps achieved the maximum score of 3 points, incorporating features such as communication with health services, including chatbots; diaries for recording signs and symptoms; and options for sharing information with healthcare professionals. Among these nine apps, 7 apps provided references to scientific literature. Considering all 32 apps analyzed, 20 provided bibliographic references.

**Table 3:** Evaluation of applications using the adapted scale *Applications Scoring System*

Code	App comprehensiveness	Literature used	In-app purchase	Connectivity	Ads	Text search field	Compatibility	Images Figures	Videos	Special features	Ease of navigation	Total Score
	Includes exchanging messages with the health service/chatbot, diary for recording signs and symptoms, information sharing	Presents literature used to support the content	In-app purchases	Internet required to work	Presence of advertisements	Search field	It is compatible with iOS and Android	Contains Images and Figures	Contains Videos	Contains user technical support or notification settings, alarms	Provides ease of navigation	
App 1	0	0	1	0	1	1	0	1	1	1	0	6
App 2	2	0	1	0	1	0	1	1	1	1	1	9
App 3	1	0	1	0	1	0	1	1	0	0	1	6
App 4	2	1	1	0	1	0	0	1	1	1	1	9
App 5	3	1	1	0	1	0	0	1	1	1	1	10
App 6	0	1	1	0	1	0	0	1	0	0	0	5
App 7	0	0	1	1	1	0	0	0	1	0	1	6
App 8	1	1	1	0	1	0	0	1	0	0	1	7
App 9	0	1	0	0	1	1	1	1	0	1	0	6
App 10	0	0	1	0	1	1	0	1	1	1	1	7
App 11	3	0	1	0	1	1	1	1	0	1	1	10
App 12	3	1	1	0	1	0	0	1	1	1	1	10
App 13	3	1	1	0	1	0	0	1	0	1	1	9
App 14	3	1	1	0	1	0	0	1	0	1	1	9
App 15	3	1	1	0	1	0	0	1	0	1	1	9
App 16	1	1	1	0	1	1	1	1	0	0	1	8
App 17	2	1	1	0	1	0	1	1	1	1	1	10
App 18	3	1	1	0	1	0	0	1	1	1	1	10
App 19	3	1	1	0	1	0	0	1	0	1	1	9
App 20	1	0	1	0	1	0	0	1	1	1	1	7
App 21	2	1	1	0	1	0	0	1	1	1	1	9
App 22	2	1	1	0	1	0	0	1	1	1	1	9
App 23	0	0	1	0	1	0	0	0	1	1	1	5
App 24	0	1	1	1	1	0	0	0	0	1	1	6
App 25	0	0	1	0	1	0	1	0	0	1	1	5
App 26	1	1	1	0	1	1	1	1	1	1	1	10
App 27	2	1	0	0	1	1	0	1	0	1	1	8
App 28	1	1	1	0	1	1	1	1	1	1	1	10
App 29	0	0	1	1	1	0	0	1	1	0	1	6
App 30	0	0	1	1	1	0	0	1	1	0	1	6
App 31	1	1	1	0	1	1	0	1	0	1	1	8
App 32	3	0	0	0	0	1	1	1	0	1	1	8

Source: Prepared by the authors and adapted from Machado, 2022

Regarding monetization, three apps offered in-app purchases and one displayed advertisement during use. Most apps required an internet connection (*n* = 28) to access the content. Functional elements included a search field (*n* = 10), images/figures (*n* = 28), and videos (*n* = 17). Additionally, 25 apps offered special features such as user support and/or notification/alarm settings, which may enhance user experience and engagement. As for usability, 29 apps were classified as easy to navigate.

## Discussion

The incorporation of digital technologies into clinical practice has intensified in both service delivery and the patient experience. In this context, the COVID-19 pandemic acted as a catalyst for the development and adoption of health technologies, including apps designed for cancer care [2]. Notably, among the 31 apps for which the release year could be identified, 23 (74.2%) were launched from 2021 onward, including 14 (45.16%) in 2024. The concentration of release dates in recent years may reflect the growing development and availability of digital tools for cancer patient education.

The analysis of 32 applications identified on the App Store and Google Play revealed a predominance of the Medicine *and* Health/Fitness categories. Most apps were designed to provide general information about various types of cancer, with a particular focus on breast cancer. They also addressed topics related to treatment, management of toxicities, self-care, and social support.

There has been a marked expansion in app development over the past decade, with a higher concentration of releases in 2023 and 2024, underscoring the recent growth of these technologies. However, only a limited number of apps included user reviews, suggesting low levels of validation among the target population. In addition, the predominance of English and the scarcity of Portuguese versions were noted, restricting access for users whose primary language is not English.

The recent growth in the development of mobile applications for cancer care is consistent with the scientific literature, which recognizes mobile technologies and other digital health interventions as promising tools to promote positive behavioral changes, support self-management, facilitate symptom monitoring, improve treatment adherence, and strengthen communication between patients and healthcare professionals, thereby complementing oncology care. These benefits have been associated with improved quality of life, reduced psychological distress, and lower utilization of emergency healthcare services, although evidence supporting their clinical effectiveness remains limited [24–26].

The findings indicate important advances but also reveal persistent gaps in scientific validation, cultural adaptation, and user engagement. These results underscore the need to develop accessible, interactive, and culturally contextualized solutions that can support health education in a reliable and evidence-based manner.

Among the analyzed applications, those targeting breast cancer were predominant, accounting for 37.5% of the sample. This concentration may be associated with global efforts to develop technological solutions for breast cancer, the most common malignancy among women [27]. The literature indicates that breast cancer is the second most frequent malignant neoplasm worldwide, with approximately 2.3 million new cases and more than 685,000 deaths annually [28]. The convergence between the present findings and published data underscores the need to invest in educational digital tools, as global projections estimate that the costs associated with breast cancer may exceed two trillion dollars by 2050 [29].

Regarding the app comprehensiveness criterion, nine (28.1%) of the 32 evaluated applications achieved the maximum score, demonstrating functionalities such as communication mechanisms with health services through chatbots, symptom monitoring diaries, and options for sharing information with health professionals. However, the limited availability of interactive features may compromise user adherence, as the absence of effective communication and collaboration channels between patients and health care teams represents a significant barrier [17, 30].

Critical content analysis revealed that several apps did not provide references to the literature used to support their content. Although the inclusion of references may indicate an attempt to ground app content in scientific evidence, it does not guarantee the accuracy, quality, or currency of the information provided. Previous research [18], reported that fewer than 20% of apps cited the sources from which their content was derived. The limited reporting of supporting references may make it more difficult for users and healthcare professionals to assess the evidence underlying the information provided.

Furthermore, the descriptions provided for most apps did not mention the involvement of researchers or healthcare professionals in their development or in the scientific validation of their content. Although the absence of this information does not necessarily indicate a lack of professional involvement, similar concerns have been documented in the literature, which identifies the absence of institutional affiliation, professional validation, and interactivity as recurrent weaknesses of such apps [17]. Recent reviews indicate that the ecosystem of mobile applications for oncology care remains fragmented, characterized by substantial variability in app quality, limited scientific underpinning, a lack of studies evaluating clinical effectiveness, and insufficient involvement of patients and healthcare professionals throughout the development process of these technologies [24]. These aspects contradict the recommendations of the World Health Organization [31], which emphasize the integration of scientific evidence and the involvement of experts from the earliest stages of technological development. In addition, it was not possible to confirm the inclusion of end users—specifically cancer patients—in the evaluation or validation phases of the available content, a limitation that may compromise the alignment of information with the target population’s actual needs.

The results of this study also indicate that accessibility remains a major barrier. Twenty-eight of the analyzed applications required continuous internet access to display their content, substantially limiting their usability in regions with inadequate infrastructure and in contexts of greater social and technological vulnerability. In 2020, a survey conducted among Medicare beneficiaries revealed that 41.4% of participants did not have a computer with broadband internet, 40.9% did not have a smartphone with wireless access, and 26.3% lacked both devices. These percentages were even higher among individuals with lower income, self-reported disability, or an educational level equal to or below high school [32, 33].

Within this accelerated digital transformation, widening inequalities in access to digital care were also observed. A North American study conducted during the pandemic found that Black and Hispanic/Latino patients, regardless of age, were more likely to avoid virtual care and instead seek in-person services. This finding reveals a paradox: although apps can expand access to information, they may also exacerbate disparities when not designed with equity considerations [34].

This scenario reveals a concerning dilemma: although digital technologies are promoted as tools to expand access to health care, their effectiveness may be limited. Exclusive reliance on digital connectivity, without consideration of accessible offline solutions, disregards the reality of a substantial portion of the population. Therefore, the development of health-focused applications must incorporate the principles of digital equity [35]. These include creating offline functionality, optimizing for low-end devices, and implementing inclusive usability strategies.

Another relevant aspect concerns the functional characteristics analyzed. Features such as internal search mechanisms, visual resources, technical user support, programmable notifications, and alarms play a crucial role in the usability and attractiveness of platforms, promoting greater user engagement with the provided content. The literature indicates that visual resources facilitate comprehension of complex information, add contextual value to the transmitted message, and help maintain user engagement during navigation [36, 37]. Moreover, responsive design and clear visual organization are recognized as important facilitators of readability and adherence to mobile technology–based interventions.

From a critical standpoint, the absence or limitation of these features can undermine the effectiveness of digital health interventions, particularly among users with limited health literacy or low technological familiarity. Investment in user-centered design and personalized experiences is therefore recommended to maximize the impact of digital tools. Apps development should consider not only the technical and scientific dimensions but also the aesthetic, functional, and communicational aspects that determine the overall success of these tools [17, 38].

Regarding usability, 29 of the evaluated applications demonstrated intuitive and accessible navigation, which may facilitate user adoption and continued use of the tool. Evidence from previous studies indicates that ease of navigation is directly associated with the user’s ability to understand, interpret, and apply health information appropriately, thereby influencing decision-making in self-care [17]. The presence of interactive features and fluid navigation not only promotes learning but also strengthens patient autonomy in managing their own health.

In summary, this study highlights the concentration of recently launched mobile applications designed to support health education for patients with cancer. The findings may inform clinical practice by helping healthcare professionals critically assess available applications based on their methodological and functional characteristics, thereby supporting more informed decisions regarding their potential use in patient education and supportive cancer care. The results may also guide researchers and developers in designing future digital health technologies by underscoring the importance of scientific validation, multidisciplinary collaboration, active user involvement, and the inclusion of features that promote accessibility, usability, and digital equity.

The findings of this study should be interpreted in light of some limitations. First, the app search strategy relied exclusively on the keywords “cancer” and “oncology.” Although these terms were directly aligned with the study objective, this approach may have limited the identification of potentially relevant apps for patients with cancer, particularly those whose app store descriptions did not explicitly include either term. Additionally, the searches were conducted in the Brazilian versions of the App Store® and Google Play®, and app availability may vary across countries and regions. Therefore, applications available exclusively in other geographic markets may not have been identified, which may limit the generalizability of our findings to the global landscape of mobile apps for cancer patient education. Second, only apps available for free download were included, potentially excluding paid apps with different characteristics or quality attributes. Third, the study focused on the characteristics, content, and functionalities of the identified apps and did not evaluate their clinical or educational effectiveness or their impact on patient outcomes. Therefore, the findings should not be interpreted as evidence of the effectiveness of individual apps or as supporting their clinical recommendation. Finally, given the dynamic nature of app stores and the frequent updating of mobile applications, the content, functionality, and availability of the included apps may have changed since the assessment was conducted.

## Conclusion

This study identified, classified, and analyzed free mobile apps for adult cancer patient education available in the App Store® and Google Play®. Most apps were launched from 2021 onward, and their content was predominantly focused on breast cancer. The evaluation also showed that not all apps provided references to the literature used to support their content, and most required an internet connection to access their content.

These findings highlight opportunities to strengthen the development of cancer education apps through greater attention to the scientific basis of their content, accessibility, and digital equity. Reducing dependence on continuous internet connectivity may improve access to educational content, particularly in settings where connectivity is limited or unstable. The involvement of patients, nurses, other healthcare professionals, researchers, and user experience specialists in the development and evaluation of these technologies should also be encouraged to promote clinically relevant, accessible, and patient-centered educational resources.

Future research should move beyond the characterization of available applications toward evaluating their usability, user experience, educational and clinical effectiveness, and long-term engagement. Studies investigating outcomes such as knowledge acquisition, treatment adherence, symptom management, and patient-reported and clinical outcomes may provide stronger evidence regarding the benefits and limitations of these technologies. Such evidence is needed to support the safe, equitable, patient-centered, and evidence-informed integration of these technologies into clinical practice.

## Data Availability

No datasets were generated or analysed during the current study.
